# Cellular crosstalk of macrophages and therapeutic implications in non-small cell lung cancer revealed by integrative inference of single-cell transcriptomics

**DOI:** 10.3389/fphar.2023.1295442

**Published:** 2023-11-15

**Authors:** Lei Wu, Wenying Xia, Yiting Hua, Kun Fan, Yanfei Lu, Min Wang, Yuexinzi Jin, Wei Zhang, Shiyang Pan

**Affiliations:** ^1^ Department of Laboratory Medicine, The First Affiliated Hospital of Nanjing Medical University, Nanjing, China; ^2^ Branch of National Clinical Research Center for Laboratory Medicine, Nanjing, China

**Keywords:** NSCLC, scRNA-seq, tumor-associated macrophages, cellular crosstalk, therapeutic implications

## Abstract

**Introduction:** Non-small cell lung cancer (NSCLC) exhibits heterogeneity with diverse immune cell infiltration patterns that can influence tumor cell behavior and immunotherapy. A comprehensive characterization of the tumor microenvironment can guide precision medicine.

**Methods:** Here, we generated a single-cell atlas of 398170 cells from 52 NSCLC patients, and investigated the imprinted genes and cellular crosstalk for macrophages. Subsequently, we evaluated the effect of tumor cells on macrophages and verified the expression of marker genes using co-culture experiments, flow cytometry and RT-qPCR assays.

**Results:** Remarkable macrophage adaptability to NSCLC environment was observed, which contributed to generating tumor-associated macrophages (TAMs). We identified 5 distinct functional TAM subtypes, of which the majority were SELENOP-positive macrophages, with high levels of *SLC40A1* and *CCL13*. The TAMs were also involved in mediating CD8^+^ T cell activity and form intercellular interaction with cancer cells, as indicated by receptor-ligand binding. Indirect coculture of tumor cells SPC-A1 and THP-1 monocytes, produced M2-like TAMs that highly expressed several markers of SELENOP-positive macrophages. The abundance of this type TAMs seemed to be associated with poorer overall survival rates [hazard ratio (HR) = 1.34, 95% confidence interval (CI) = 0.98-1.83, *p* = 0.068] based on deconvolution of TCGA-LUAD dataset.

**Discussion:** In summary, we provided a high-resolution molecular resource of TAMs, and displayed the acquired properties in the tumor microenvironment. Dynamic crosstalk between TAMs and tumor cells via multiple ligand-receptor pairs were revealed, emphasizing its role in sustaining the pro-tumoral microenvironment and its implications for cancer therapy.

## Introduction

Lung cancer is one of the most common cancers worldwide, and it remains the leading cause of cancer-related deaths (Sung et al., 2021). Although immune checkpoint inhibitors have shown remarkable efficacy in treating non-small cell lung cancer (NSCLC), only a fraction of patients respond to these therapies (Zhang Y. et al., 2023; Lin et al., 2023). Identifying the factors that contribute to the development and progression of lung cancer is critical for improving patient outcomes. In recent years, single-cell sequencing (scRNA-seq) technology has been utilized to depict the immune microenvironment of NSCLC, which offered insight into the complex interactions between tumor cells and immune cells, primarily mediated through cytokines and chemokines (Wang et al., 2022; Pai et al., 2023). This technology allows for a granular analysis, uncovering diverse cell populations within the tumor ecosystem and their molecular features, shedding light on potential therapeutic targets.

Tumor-associated macrophages (TAMs) are an essential component of the tumor microenvironment (TME) surrounding cancer cells. TAMs exhibit a heterogeneous population of macrophages that are derived from tissue-resident macrophages, monocytes, and bone marrow-derived progenitor cells. They accumulate progressively during disease progression, with high densities of TAMs being associated with poor prognosis in lung cancer patients (Garrido-Martin et al., 2020; Yoshida et al., 2021). The polarization of TAMs has been studied extensively, and two main phenotypes have been described: M1 and M2. M1 macrophages produce pro-inflammatory cytokines, involved in tumor cell killing, whereas M2 macrophages release anti-inflammatory cytokines and promote tumor growth and angiogenesis (Zhang H. et al., 2021). The function of airway macrophages can be epigenetically regulated and resulting transcriptomic and phenotypical changes (Hey et al., 2021). The majority of scRNA-seq studies have reported that the phenotype of TAMs is plastic and can be reprogrammed, contributing to exert a mixture of phenotypic characteristics (Kim et al., 2020). Targeting these modified macrophages could potentially offer therapeutic avenues for patients with lung diseases.

Recent advances in tumor biology have revealed that the differentiation and intricate communication of TAMs can be leveraged as a promising target for the development of effective therapies aimed at disrupting critical interactions within the tumor microenvironment. Ruella *et al.* reported that immunosuppressive M2-type TAM expressed high levels of CD123 in Hodgkin lymphoma TME, and anti-CD123 chimeric antigen receptor (CAR) T cells could recognize and kill TAMs thereby overcoming immunosuppression, representing a promising new therapeutic approach (Ruella et al., 2017). Sánchez-Paulete and colleagues developed CAR-T cells targeting macrophages to achieve significant antitumor efficacy and reprogram the immunosuppressive TME in mouse models of lung, ovarian, and pancreatic cancer (Sánchez-Paulete et al., 2022). Liang *et al.* engineered a macrophage-mediated cellular phagocytosis-boosting hydrogel that reshaped the TME, leading to the acceleration of TAMs’ polarization into the anti-tumoral M1-like phenotype and the initiation of tumor-specific CD8^+^ T cell responses (Liang et al., 2023).

**FIGURE 1:**
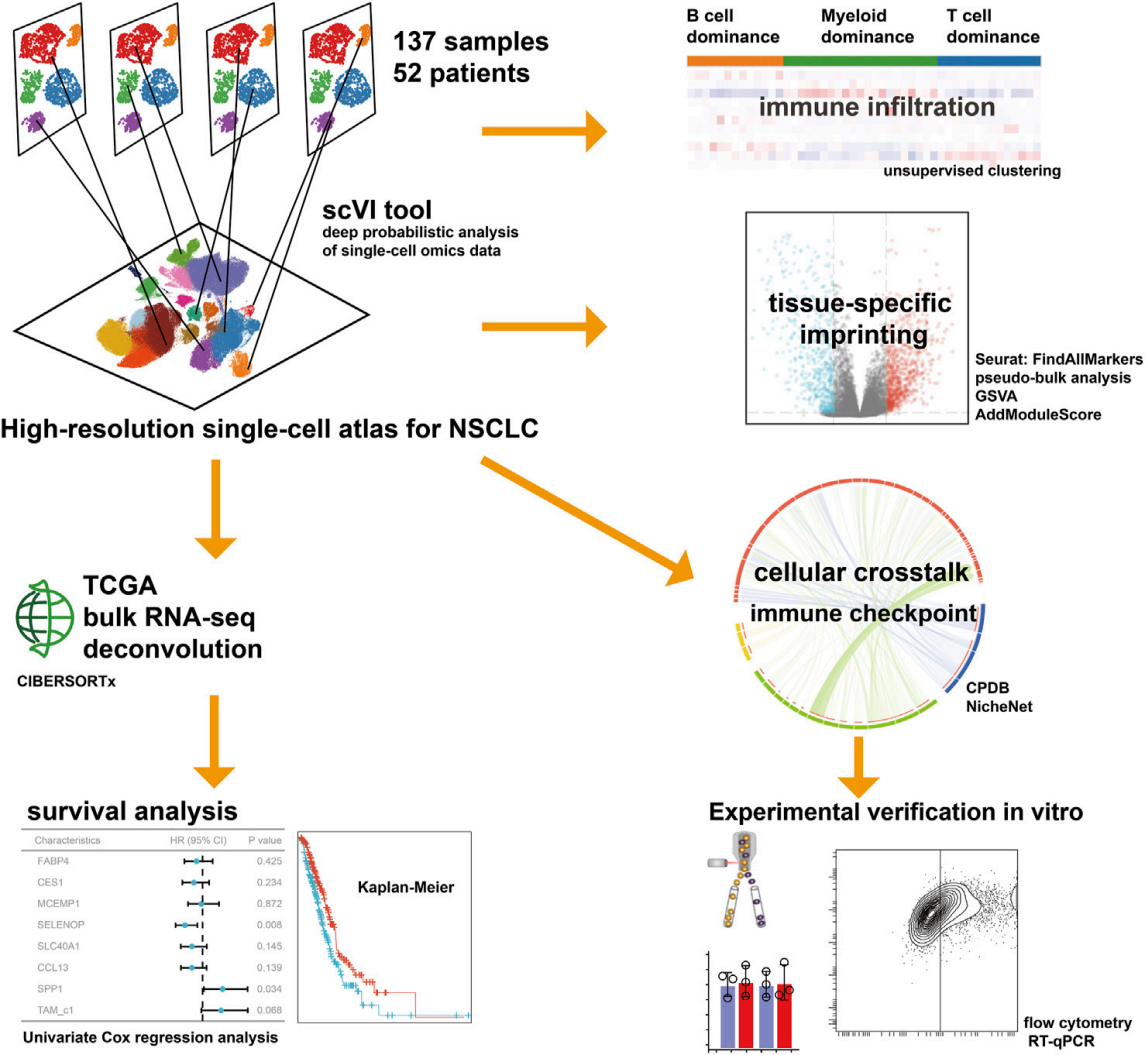
The workflow of the present study.

In the present study, we conducted an integrative inference of previously published scRNA-seq data to profile the TME of NSCLC, characterizing the interconvertibility and interaction among different types of macrophages, with a particular focus on the cellular crosstalk of TAMs. We reported the comprehensive single-cell transcriptome profiling of NSCLC covering 52 patients, and unveiled cellular dynamics and molecular features associated with the tumor progression for TAMs, thus extending our understanding of adaptive immune system. Deciphering the foundational cellular mechanisms and orchestrating these interactions holds the potential approach for indirectly impeding the interplay among cancer cells. This novel avenue could significantly advance the creation of effective and secure therapeutic strategies in the battle against cancer. The workflow for this study is depicted in Figure 1.

## Methods

### Single-cell RNA-seq datasets collected in this study

The scRNA-seq data of NSCLC used in this study were downloaded from website (http://lungcancer.chenlulab.com(Lambrechts et al., 2018; Zhang et al., 2022), https://doi.org/10.24433/CO.0121060.v1(Bischoff et al., 2021). To ensure the stability and comparability of integration, scRNA-seq datasets based on the 10x Genomics Chromium platform were included. Cell annotation tables along with quality control metrics were obtained from the original publications. The quality-passed cells were used for downstream analysis.

### Normalization and integration of scRNA-seq data

Cells with fewer than 200 genes detected or > 40% mitochondrial counts or > 50% ribosomal counts were removed for following analysis; genes detected in > 3 cells and with unique molecular identified (UMI) count > 1,000 were kept. We ran the Scrublet algorithm to eliminate any potential doublets, setting the expected doublet rate to 0.05 (Wolock et al., 2019). Subsequently, individual datasets were merged into a single AnnData object, and then were integrated using the scVI algorithm. This algorithm has demonstrated its capability as one of the top-performing methods for integrating atlas-level data and scaling to over one million cells (Lopez et al., 2018). Two-dimension Uniform Manifold Approximation and Projection (UMAP) embeddings and unsupervised Leiden-clustering with scanpy were computed based on a cell-cell neighborhood graph derived from scVI latent space (Becht et al., 2018). Cell clusters in the resulting two-dimensional representation were annotated to known biological cell types using canonical marker genes.

### Identification of differentially expressed genes

We applied the Wilcoxon Rank-Sum test in FindAllMarkers function to identify differentially expressed genes for each cell clusters, with the following parameters: only considering positive markers, fraction of expressing cells inside the cluster to be ≥ 0.25, log fold change between cells inside and outside the cluster to be ≥ 0.25. Based on the subpopulation-stratified scRNA-Seq data, pseudo-bulk samples were created for differential expression assessment, and samples with fewer than 10 cells were removed.

### Scoring samples for M1/M2 polarization signatures

To comprehend the characteristics of distinct macrophage subsets, scores for M1/M2 polarization were acquired utilizing the AddModuleScore function within the “Seurat” package. M1 and M2 gene profiles were obtained from the study conducted by Azizi *et al* (Azizi et al., 2018).

### Receptor–ligand communication between cell types

We used the cellphonedb (CPDB) database to investigate differences in cell-to-cell communication (Efremova et al., 2020), and identified differentially expressed signaling molecules through this analysis (Salcher et al., 2022). Furthermore, using NicheNet, we additionally identified receptor-ligand interactions that are likely to have an impact on particular gene-expression changes in a target cell lineage with the prior knowledge on signaling and gene regulatory networks (Browaeys et al., 2020).

### Estimating cell-type proportions in the lung adenocarcinoma bulk RNA-seq from LUAD TCGA

The level 3 RNA-sequencing data (HTseq counts) along with metadata were downloaded from the TCGA (The Cancer Genome Atlas), specifically by selecting “lung adenocarcinoma (LUAD)” in the GDC data portal (portal.gdc.cancer.gov). Bulk RNA-seq counts were converted to transcripts per million (TPM) and matrices were deconvoluted with CIBERSORTx using scRNA-seq annotations (Newman et al., 2019). The reference matrix was created by randomly downsampling each cell-type to a maximum of 500 cells, and then utilized to generate a signature matrix file.

The TCGA-LUAD dataset comprised 530 valid cases, with a median overall survival time of 50.0 months, and out of these, 188 patients died. The prognostic significance of macrophage marker expression and percentage of macrophage subpopulation for overall survival was assessed using Kaplan-Meier curves. Univariate Cox regression analysis was applied to calculate hazard ratios (HRs) and their corresponding 95% confidence intervals (CIs).

### Cell culture and polarization of THP-1

The human lung cancer cell line SPC-A1 and human monocyte-like cells (THP-1) were purchased from the Chinese Academy of Sciences, China. Cells were cultured in RPMI 1640 medium (Gibco, USA), and supplemented with 10% fetal bovine serum (FBS) and 1% penicillin/streptomycin. After THP-1 cells were treated with phorbol 12-myristate 13-acetate (PMA, 180 ng/ml) for 24 h, the cells differentiated into macrophages, and then co-cultured with SPC-A1. At termination, tumors were excised, and single-cell suspensions were prepared, and then stained with anti-CD68-APC and anti-CD163-PE (BioLegend) and analyzed by flow cytometry.

### Reverse transcription quantitative polymerase chain reaction (RT-qPCR)

Total RNA was extracted from cells using TRIzol reagent (Invitrogen, USA; 15596026), and Primescript RT Reagent Kit (TaKaRa, Japan; RR036A) was used for reverse transcription. A 7500 Real-Time PCR System (Applied Biosystems, USA) was applied for RT-qPCR using SYBR Premix Ex Taq Kit (TaKaRa, Japan; RR091A). The primers used for amplification were listed in Supplementary Table S1.

### Quantification and statistical analysis

Utilizing the Wilcoxon Rank-Sum test or the two-sided Student’s t-test, differential analysis was conducted between the two groups. The Kruskal-Wallis test was used to analyze differences among multiple pairwise comparisons. *p*-values under 0.05 were regarded as significant.

## Results

### Transcriptomic characterization of the TME in NSCLC by comprehensively integrating scRNA-seq data

**FIGURE 2:**
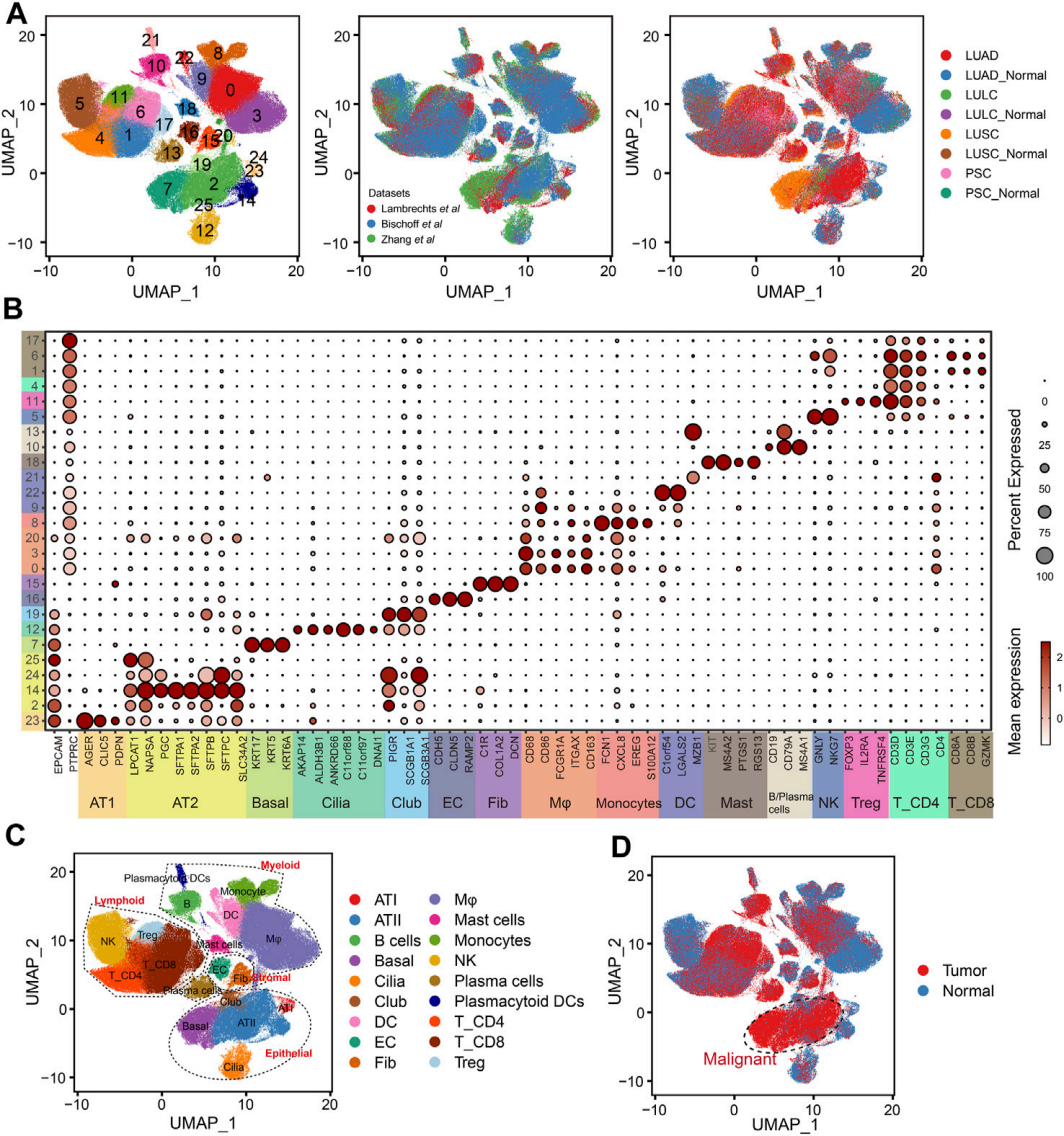
Integration of NSCLC from three scRNA-seq datasets. **(A)** UMAP plots showing the integration of three scRNA-seq datasets by scVI tool. **(B)** Dot plot displaying average and percent expression of marker genes for clusters in Figure 2A. UMAP plots showing the overview of NSCLC atlas colored by **(C)** major cell types, and **(D)** tissue type.

In order to depict the TME of NSCLC, we integrated and analyzed scRNA-seq data from three studies comprising 137 samples and 52 patients (Supplementary Table S2). The scVI tool was used to remove the batch effect, which yielded a total of 398170 cells. After quality control, normalization, identification of most variable genes, and dimensionality reduction, UMAP analyses created 26 unsupervised clusters (Figure 2A). Based on the expression levels of cell-type-specific markers, we identified 18 major cell populations, including alveolar type I cells (AT1, marked with *AGER*, *CLIC5* and *PDPN*), alveolar type II cells (AT2, marked with *LPCAT1*, *NAPSA*, *PGC* and *SLC34A2*), basal cells (Basal, marked with *KRT17*, *KRT5* and *KRT6A*), ciliated cells (Cilia, marked with *AKAP14*, *ALDH3B1*, *ANKRD66*, *C11orf88*, *C11orf97* and *DNAI1*), club cells (Club, marked with *PIGR*, *SCGB1A1* and *SCGB3A1*), endothelial cells (EC, marked with *CDH5*, *CLDN5* and *RAMP2*), fibroblasts (Fib, marked with *C1R*, *COL1A2* and *DCN*), macrophages (Mφ, marked with *CD68*, *CD86*, *FCGR1A*, *ITGAX* and *CD163*), Monocytes (marked with *FCN1*, *CXCL8*, *EREG*, *S100A12*), dendritic cells (DC, marked with *C1orf54*, *LGALS2* and *MZB1*), mast cells (Mast, marked with *KIT*, *MS4A2*, *PTGS1* and *RGS13*), B cells and plasma cells (marked with *CD19*, *CD79A* and *MS4A1*), natural killer cells (NK, marked with *GNLY* and *NKG7*), regular T cells (Tregs, marked with *FOXP3*, *IL2RA* and *TNFRSF4*), CD4^+^ T cells (T_CD4, marked with *CD3D*, *CD3E*, *CD3G* and *CD4*) and CD8^+^ T cells (T_CD8, marked with *CD8A*, *CD8B* and *GZMK*, Figures 2B, C). Based on the tissue of origin, epithelial cell compartments were classified as normal or malignant cell clusters (Figure 2D), which was mainly consistent with the copy-number status of cells (Supplementary Figure S1). Besides, the dominant malignant cells in NSCLC were AT2 and basal cells.

### Characterization of the NSCLC immune cell atlas

**FIGURE 3:**
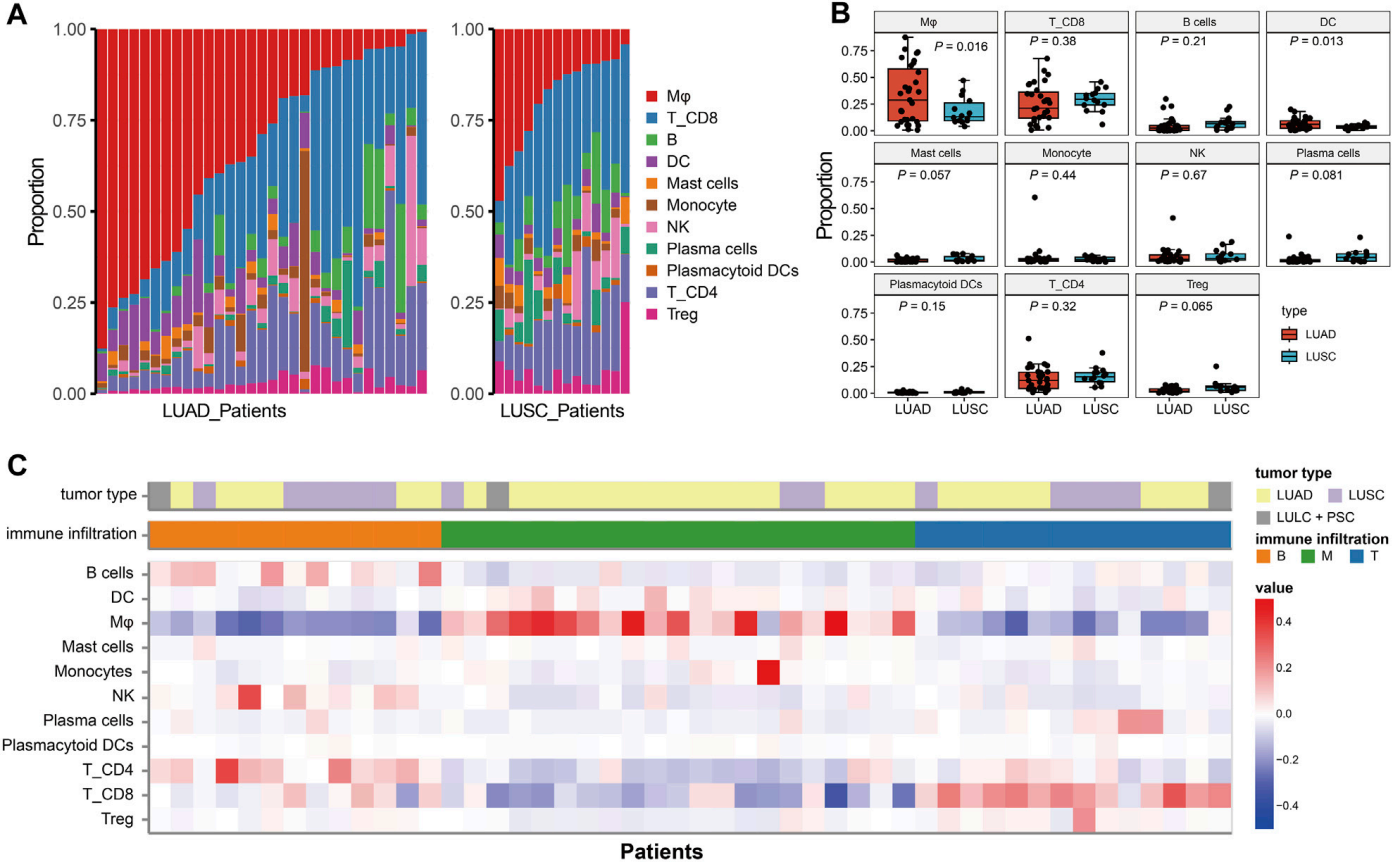
The immune infiltration phenotypes in NSCLC based on scRNA-seq. **(A)**: Proportion of each immune cell type in LUAD and LUSC patients. **(B)** Bar plots represent the distribution of eleven immune cell types between LUAD and LUSC. **(C)** Characteristics of the NSCLC patients and classification of the tumor immune phenotypes.

Single-cell immune profiling could accurately reveal the contribution of the TME, making it possible to detect diverse immune cell compositions within different cancer types. The proportion of eleven immune cell lineage varied between LUAD and LUSC (Figure 3A), revealing a heterogeneous cellular status. In addition, we found that the frequency of macrophages was significant higher in LUAD than LUSC (*p* = 0.016, Figure 3B). Salcher *et al.* have revealed four distinct tumor immune phenotypes in NSCLC, (i) immune-desert (ID); (ii) B cell dominance (B); (iii) myeloid dominance (M); and (iv) T cell dominance (T) (Salcher et al., 2022). As shown in Figure 3C, correlating immune cell population frequencies in NSCLC identified an intriguing phenomenon that patients with LUAD had high infiltration of the macrophages (the subtype of tumors with myeloid dominance, M).

### Diversity within macrophage subsets in NSCLC

**FIGURE 4:**
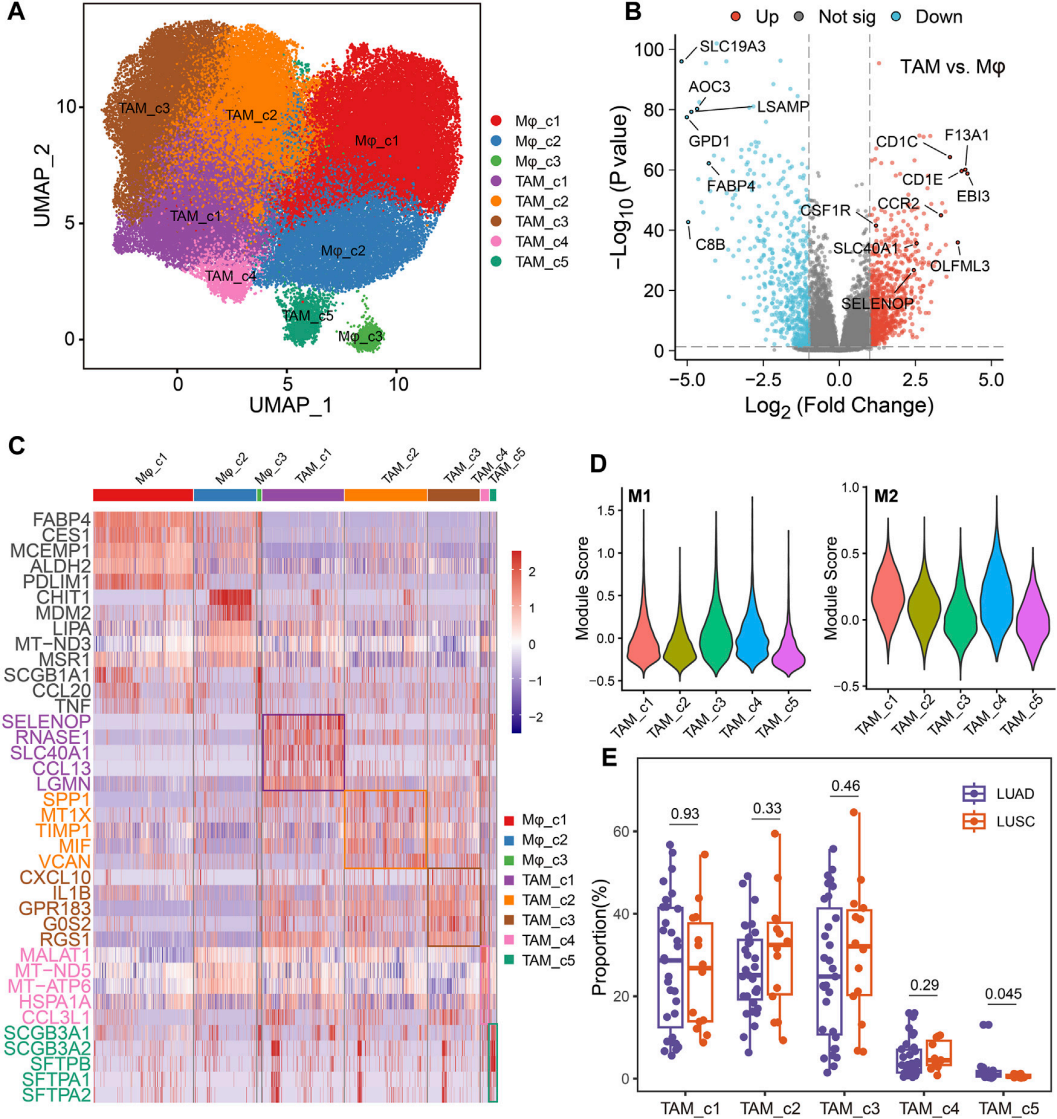
Dynamic restructuring of macrophages in NSCLC. **(A)** UMAP plots showing the macrophages in NSCLC, classified into tumor-associated macrophages (TAMs) and normal-adjacent associated macrophages (Mφ). **(B)** Volcano plot of differentially expressed genes between TAMs and Mφs. **(C)** Heatmap showing the differentially expressed genes (rows) across different macrophage clusters (columns). **(D)** Module scores of gene signatures related to M1/M2 polarization across different TAM clusters. **(E)** Boxplot showing cellular fractions of TAM clusters in 31 LUAD and 14 LUSC patients. One-sided unpaired Wilcoxon test was used.

TAMs have diverse functions in cancer with both anti- and pro-tumorigenic properties (Ma et al., 2022). Analysis of the single-cell transcriptomic profile of 91260 macrophage cells, revealed 8 distinct clusters, including 3 normal macrophage clusters and 5 TAM clusters (Figure 4A). Compared to normal macrophage, TAMs highly expressed *EBI3* which is subunit of the composite cytokines IL-27 and IL-35 and monocyte differentiation markers, such as *F13A1*, *CCR2* and *CSF1R* (Figure 4B). As shown in Figure 4C, TAM_c1 cluster was characterized by a high expression of *SELENOP* and *CCL13*, that have been related to M2 polarization (Solinas et al., 2010; Sathe et al., 2020). Meanwhile, *SLC40A1* is highly expressed on this subset where it mediates iron efflux from the breakdown of haem. TAM_c2 cluster displayed high expression of *SPP1*, which was reported to mediate macrophage polarization and associated with an immunosuppressive TME (Zhang et al., 2017). *TIMP1* was significantly elevated in this subcluster, that play a crucial role in extracellular matrix regulation (Ishihara et al., 2023). There is an upregulation of M1 polarized markers (proinflammatory cytokines *CXCL10* and *IL1B*) in the TAM_c3 subcluster. TAM_c4 showed high expression of mitochondrial genes reflecting enhanced ATP metabolism. Finally, we observed TAM_c5 subpopulation displaying high expression of LUAD markers such as *SCGB3A1*, *SCGB3A2* and *SFTPB*, that seemed to have anti-inflammatory effects and anti-fibrotic activity in lung (Cai et al., 2014). The transcriptional profiles of the TAM subsets indicated their heterogeneity and plasticity. Gene set variation analysis (GSVA) of hallmark pathways revealed there were increased activities of inflammatory response and glycolysis in TAMs (Supplementary Figure S2). We further assessed the M1, and M2 signatures of different TAM subsets. The classical M1/M2 model could not completely explain the polarization of macrophages, and both M1 and M2 associated genes frequently expressed in the same subsets. TAM_c1, c2 and c4 subsets showed high anti-inflammatory M2 scores, and TAM_c3 subset had high pro-inflammatory M1 score (Figure 4D). We further calculated the fractions of all TAM clusters in 31 LUAD and 14 Lung squamous cell carcinoma (LUSC) patients. The distribution of TAM c1-c4 clusters was not significant between LUAD and LUSC, whereas a higher fraction of TAM_c5 were observed in LUAD (Figure 4E).

### Cellular crosstalk in the TME

**FIGURE 5:**
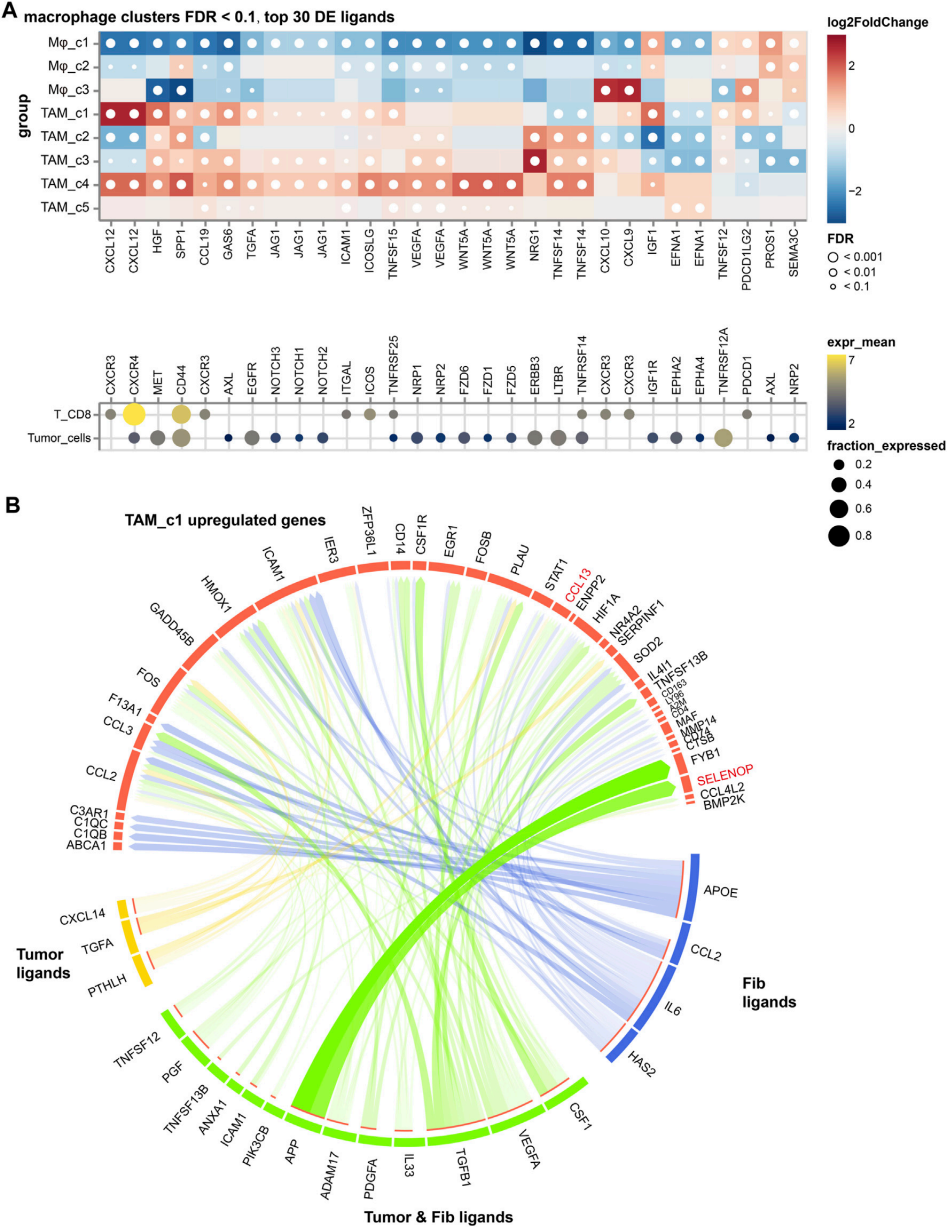
The cellular crosstalk of macrophages in NSCLC. **(A)** Outgoing interactions of different macrophage subclusters with cancer cells and CD8^+^ T cells. **(B)** Predicted ligands in tumor cells and fibroblasts that regulate respective target genes in TAM_c1 cluster.

To explore the contribution of TAM subsets in NSCLC, we analyzed the cellular interactions between TAMs with cancer cells and CD8^+^ T cells, based on the differentially expressed TAM ligands [False Discovery Rate (FDR) < 0.01, absolute log2FC > 1]. As shown in Figure 5A, we discovered VEGFA signaling toward cancer cells in all TAM subsets, highlighting its significant proangiogenic significance. Enhancement of the SPP1 signaling was observed in the TAM_c1, c2 and c4 subsets and suppressed T cell activation, which partially explained their high M2 scores. Expression of *CXCL12* is highest in TAM_c1 subset, supporting revascularization of ischemic tissue and tumor growth (Teicher and Fricker, 2010). We further used the NicheNet algorithm to identify intercellular communication of tumor cells that might influence the transcriptional phenotype of TAMs. Interestingly, we found PTHLH derived from tumor cells can target macrophages and regulate the expression of *CCL13*, *PLAU* and *ICAM1*, resulting the phenotype of TAM_c1. Ligand APP from tumor cells and fibroblasts might increase the expression of *SELENOP* in macrophages (Figure 5B).

**FIGURE 6:**
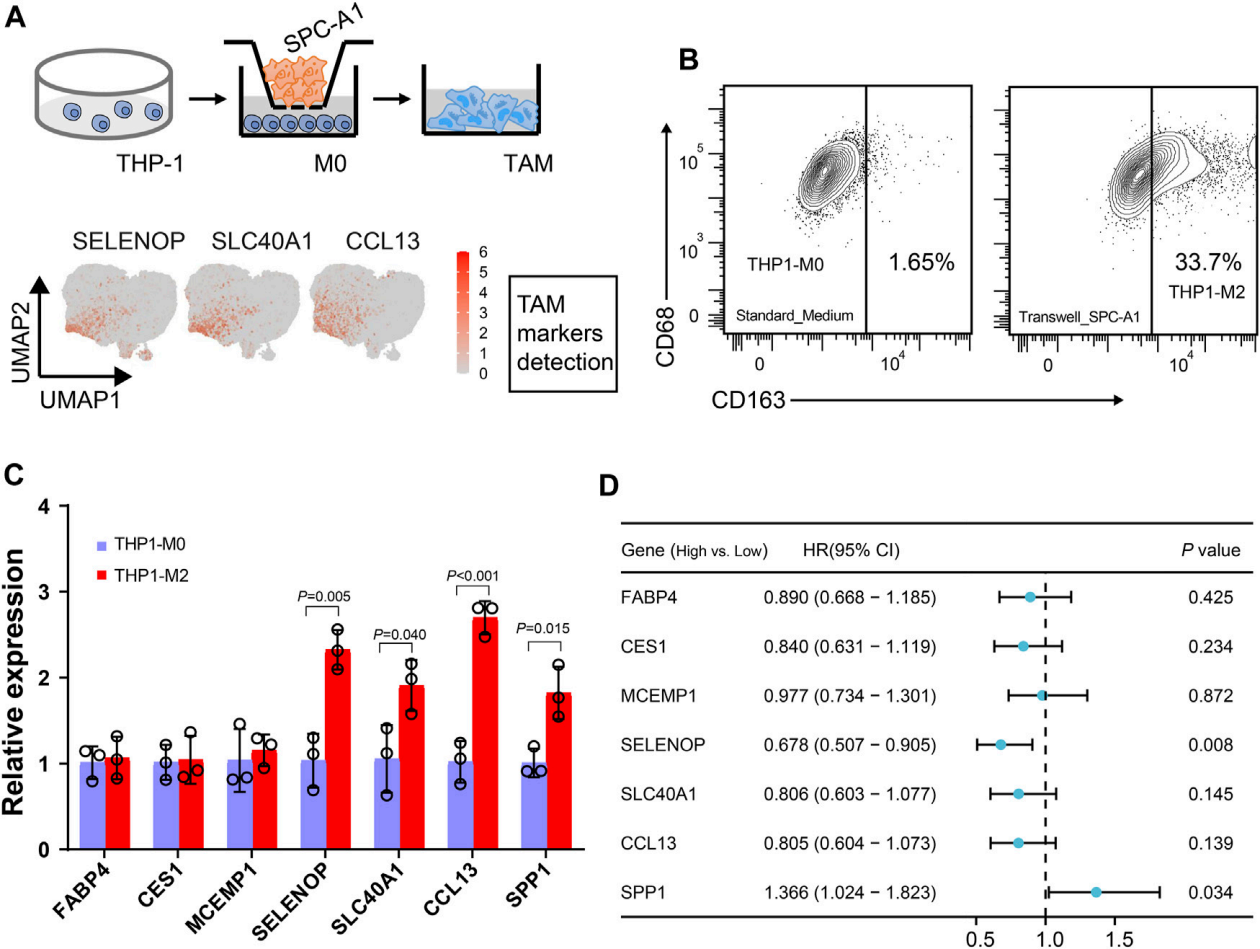
Dissection of the interaction between macrophages and tumor cells. **(A)** Workflow depictingTHP-1 monocyte-derived macrophages that were co-cultured with SPC-A1. **(B)** Flow cytometry analysis of M2-like macrophages. **(C)** Boxplot showing the mean expression of the marker genes of Mφ_c1 and TAM_c1 clusters for M2-like macrophages. **(D)** Univariate Cox regression analysis of macrophage markers expression and TAM_c1 abundance associated with the survival in LUAD patients. LUAD patients were stratified into high and low groups based on the median TPM (transcripts per million) values of the analyzed genes, as well as the median TAM_c1 abundance.

While five tumor-associated macrophage (TAM) subtypes are present in both LUAD and LUSC, macrophages are notably more abundant in adenocarcinoma. Furthermore, we used *in vitro* transwell co-culture experiment to verify the certain cytokine secreted by tumors could induce THP-1 monocyte differentiated into the state in the LUAD TME (Figure 6A). When THP-1 monocytes co-cultured with SPC-A1 cells, about 33.7% THP-1 could be successfully polarized into M2 macrophages (THP1-M2, Figure 6B). Importantly, the expression of *SELENOP*, *SLC40A1*, *CCL13* and *SPP1* in the macrophages were significantly upregulated in THP1-M2, while *FABP4*, *CES1* and *MCEMP1* displayed slight variation during the induction (Figure 6C).

Using clinical data collected from TCGA, we next evaluated the impact of these TAM marker genes on prognosis. Forest plot from univariate regression analysis demonstrated that LUAD patients with high levels of *SPP1* were related to poor prognosis (Figure 6D, Supplementary Figure S3A), whereas high *SELENOP* expression indicated favor prognosis (Figure 6D, Supplementary Figure S3B). Focusing on the clinical relevance of TAM_c1 in LUAD, we utilized CIBERSORTx to estimate the proportion in TCGA dataset based on the cell-type markers in scRNA-seq. Estimates of the TAM_c1 showed high intra-group correlation within other TAM groups (TAM_c2-4, Supplementary Figure S4A), and *CCL13* was included in the signature matrix for TAM_c1 deconvolution (Supplementary Figure S4B). Importantly, TAM_c1 abundance seemed to be associated with poorer overall survival rates (HR = 1.34, 95% CI = 0.98-1.83), but the difference was not statistically significant (*p* = 0.068, Figure 6D, Supplementary Figure S3C).

## Discussion

Some clinical studies have demonstrated that decreasing the number of TAMs might be an effective tumor treatment. Hu et al. reported that tissue-resident macrophages were expanded after neoadjuvant immunotherapy in NSCLC, and M2-TAMs were more likely remodeled into a neutral (M0) instead of an anti-tumor phenotype (M1) (Hu et al., 2023). Francisco-Cruz et al. found that a higher level of infiltration of PD-L1^+^ macrophages (CD68^+^PD-L1^+^) that were in closer proximity to malignant cells in the NSCLC was associated with poor overall survival (Francisco-Cruz et al., 2023). Backman et al. investigated the spatial immunophenotyping of TME for NSCLC using multiplex immunofluorescence staining. They found that the high densities of M1 and CD163 macrophages exhibited a positive prognostic influence, whereas short M2–M1 distances were prognostically unfavourable (Backman et al., 2023). Therefore, identification of targets that can prevent M2-like TAM transition is a crucial task for future research.

In our study, Mφ_c1 cells are tissue-resident alveolar macrophages with the expression of the canonical markers (*FABP4* and *MCEMP1*). FABP4 is a member of the fatty acid-binding protein family and plays important functions in inflammation and metabolism (Xu et al., 2015). We also found that this cluster showed activity related to fatty acid metabolism, which is an accessory characteristic of alveolar macrophages. Moreover, macro_FABP4 alveolar macrophages were reported to be significantly elevated in post-treatment NSCLC patients who received neoadjuvant PD-1 blockade combined with chemotherapy (Hu et al., 2023). This phenomenon indicated that alveolar macrophages might work together with AT2 cells, being involved in alveolar epithelial regeneration. TAM_c1 showed high expression of *SELENOP*, which was previously reported to have an anti-inflammation role (Barrett et al., 2015). This cluster also highly expressed *LGMN* and *HLA-DP*, *DQ*, *DR*, indicating their intimate correlation with antigen processing and presentation. The JAK-STAT signaling shows dynamic activity in TAM_c1 and c3 clusters that is essential for a wide range of cytokines such as CCL13, CXCL10 and IL1B, leading to critical cellular events, such as hematopoiesis. Besides, the TAM_c2 cluster highly expressed *SPP1* and demonstrated maximum activity in glycolysis, which produces adenosine triphosphate (ATP) and carbon intermediates to facilitate TAM reprogramming (Zhang Q. et al., 2021). Enhanced glycolysis in TAMs supported several metabolic pathways and regulated cell signaling to promote tumor development (Zhang X. et al., 2023). Matsubara et al. found that SPP1 was highly expressed in immunologically “hot” areas such as CD163-positive TAMs, which predicted a poor prognosis in LUAD, but this association did not hold for LUSC. Additionally, they found that macrophage-derived SPP1 suppresses the apoptosis of cancer cells when exposed to anticancer drugs (PTX or PEM) (Matsubara et al., 2022).

We have also demonstrated molecular interactions between the tumor cells and immune compartments. Several significant interactions (such as SPP1-CD44, CXCL12-CXCR4 and PDCD1LG2-PDCD1) were inferred among TAMs, tumor cells and CD8^+^ T cells, which involved the activation of ERK, TGF-β and NF-κB signaling pathways in tumor cells and the negative regulation of activated T cell proliferation. Besides, we quantified fully polarized THP-1-derived M2-type macrophages using flow cytometry based on CD68 and CD163, and validated the expression of *SELENOP*, *SLC40A1*, *CCL13* and *SPP1*. It is particularly noteworthy that cell-based cancer immunotherapies hinge on the capacity of natural or engineered receptors present on immune cells to interact with specific antigens on cancer cells, resulting in the induction of tumor cell destruction. The combination of CXCR4 therapeutic agent blockade and PDCD1 resulted in the reduction of suppressive leukocytes and promoted the transition of M2-to-M1 macrophage polarization within the tumor (Pei et al., 2023). Martinez-Usatorre et al. reported that both TAMs derived from monocytes, reliant on CSF1R, and alveolar-origin TAMs, sensitive to cisplatin, played a pivotal role in shaping TME enriched in TGF-β, which facilitated the presence of PD-1^+^ Tregs. The concurrent targeting of TAMs through a combination of a CSF1R inhibitor and cisplatin diminished Tregs, redirected the function of PD-1 antibodies toward CD8^+^ T cells, and bolstered the efficacy of antiangiogenic immunotherapy, resulting in significant tumor regression (Martinez-Usatorre et al., 2021). Hence, exploring cellular dynamic crosstalk can serve as a novel strategy to indirectly disrupt the interplay of cancer cells, which contributing to the development of efficient and safe therapeutic strategies for combating cancer.

By integrating bulk and single-cell RNA sequencing, high levels of TAM_c1 subset seemed to be correlated with poor survival for lung cancer patients. Deconvolution typically relies on a set of known marker genes, that are expressed at different levels in different cell types (Erdmann-Pham et al., 2021). While certain marker genes demonstrated discernible prognostic potential, the process of deconvoluting bulk transcriptome data to estimate the relative proportions of various cell types remained unaffected. This disparity may lead to incongruities in prognostic implications between marker genes and TAM subgroups. Besides, we have observed a certain degree of correlation among different TAM subtypes based on single-cell technology, which could introduce some error when inferring the expression of individual TAM subgroups. Therefore, a more comprehensive understanding of the regulatory mechanisms governing different macrophage subsets is imperative for future research.

## Conclusion

In summary, our study generates an intricate high-resolution portrait of TME in NSCLC with 18 major cell types. Notably, we elucidate distinct cell-type composition patterns within LUAD and LUSC, providing more precise functional transcriptomic classification of macrophages in both histotypes. The biology of TAMs presented in this study could provide the theoretical basis for developing immune-checkpoint therapies for patients with NSCLC.

## Figures and Tables

**Table:** 

**NSCLC**	Non-small cell lung cancer
**LUAD**	Lung adenocarcinoma
**LUSC**	Lung squamous cell carcinoma
**TAM**	Tumor-associated macrophages
**TME**	Tumor microenvironment
**scRNA-seq**	Single-cell sequencing
**CAR**	Chimeric antigen receptor
**UMAP**	Uniform Manifold Approximation and Projection
**TCGA**	The Cancer Genome Atlas
**CD123**	Interleukin 3 Receptor Subunit Alpha
**AGER**	Advanced Glycosylation End-Product Specific Receptor
**CLIC5**	Chloride Intracellular Channel 5
**PDPN**	Podoplanin
**LPCAT1**	Lysophosphatidylcholine Acyltransferase 1
**NAPSA**	Napsin A Aspartic Peptidase
**PGC**	Progastricsin
**SLC34A2**	Solute Carrier Family 34 Member 2
**KRT17**	Keratin 17
**KRT5**	Keratin 5
**KRT6A**	Keratin 6A
**AKAP14**	A-Kinase Anchoring Protein 14
**ALDH3B1**	Aldehyde Dehydrogenase 3 Family Member B1
**ANKRD66**	Ankyrin Repeat Domain 66
**C11orf88**	HOATZ Cilia And Flagella Associated Protein
**C11orf97**	Chromosome 11 Open Reading Frame 97
**DNAI1**	Dynein Axonemal Intermediate Chain 1
**PIGR**	Polymeric Immunoglobulin Receptor
**SCGB1A1**	Secretoglobin Family 1A Member 1
**SCGB3A1**	Secretoglobin Family 3A Member 1
**CDH5**	Cadherin 5
**CLDN5**	Claudin 5
**RAMP2**	Receptor Activity Modifying Protein 2
**C1R**	Complement C1r
**COL1A2**	Collagen Type I Alpha 2 Chain
**DCN**	Decorin
**CD68**	CD68 Molecule
**CD86**	CD86 Molecule
**FCGR1A**	Fc Gamma Receptor Ia
**ITGAX**	Integrin Subunit Alpha X
**CD163**	CD163 Molecule
**FCN1**	Ficolin 1
**CXCL8**	C-X-C Motif Chemokine Ligand 8
**EREG**	Epiregulin
**S100A12**	S100 Calcium Binding Protein A12
**C1orf54**	Chromosome 1 Open Reading Frame 54
**LGALS2**	Galectin 2
**MZB1**	Marginal Zone B And B1 Cell Specific Protein
**KIT**	KIT Proto-Oncogene, Receptor Tyrosine Kinase
**MS4A2**	Membrane Spanning 4-Domains A2
**PTGS1**	Prostaglandin-Endoperoxide Synthase 1
**RGS13**	Regulator Of G Protein Signaling 13
**CD19**	CD19 Molecule
**CD79A**	CD79a Molecule
**MS4A1**	Membrane Spanning 4-Domains A1
**GNLY**	Granulysin
**NKG7**	Natural Killer Cell Granule Protein 7
**FOXP3**	Forkhead Box P3
**IL2RA**	Interleukin 2 Receptor Subunit Alpha
**TNFRSF4**	TNF Receptor Superfamily Member 4
**CD3D**	CD3 Delta Subunit Of T-Cell Receptor Complex
**CD3E**	CD3 Epsilon Subunit Of T-Cell Receptor Complex
**CD3G**	CD3 Gamma Subunit Of T-Cell Receptor Complex
**CD4**	CD4 Molecule
**CD8A**	CD8 Subunit Alpha
**CD8B**	CD8 Subunit Beta
**GZMK**	Granzyme K
**EBI3**	Epstein-Barr Virus Induced 3
**F13A1**	Coagulation Factor XIII A Chain
**CCR2**	C-C Motif Chemokine Receptor 2
**CSF1R**	Colony Stimulating Factor 1 Receptor
**SELENOP**	Selenoprotein P
**CCL13**	C-C Motif Chemokine Ligand 13
**SLC40A1**	Solute Carrier Family 40 Member 1
**SPP1**	Secreted Phosphoprotein 1
**TIMP1**	TIMP Metallopeptidase Inhibitor 1
**CXCL10**	C-X-C Motif Chemokine Ligand 10
**IL1B**	Interleukin 1 Beta
**SCGB3A1**	Secretoglobin Family 3A Member 1
**SCGB3A2**	Secretoglobin Family 3A Member 2
**SFTPB**	Surfactant Protein B
**PLAU**	Plasminogen Activator, Urokinase
**ICAM1**	Intercellular Adhesion Molecule 1
**FABP4**	Fatty Acid Binding Protein 4
**CES1**	Carboxylesterase 1
**MCEMP1**	Mast Cell Expressed Membrane Protein 1
**LGMN**	Legumain
**CXCL12**	C-X-C Motif Chemokine Ligand 12
**CXCR4**	C-X-C Motif Chemokine Receptor 4
**PDCD1LG2**	Programmed Cell Death 1 Ligand 2
**PDCD1**	Programmed Cell Death 1

## Data Availability

The original contributions presented in the study are included in the article/Supplementary Material, further inquiries can be directed to the corresponding author.

## Supplementary material

SUPPLEMENTARY TABLE S1 Table showing the list of primer sequences for RT-qPCR.
