# Supplementary figures and images for: Cellular crosstalk of macrophages and therapeutic implications in non-small cell lung cancer revealed by integrative inference of single-cell transcriptomics
Source: Front Pharmacol. 2023 Nov 15;14:1295442. doi: 10.3389/fphar.2023.1295442 (PMC10690610; doi:10.3389/fphar.2023.1295442)

**Supplementary Figure 1**


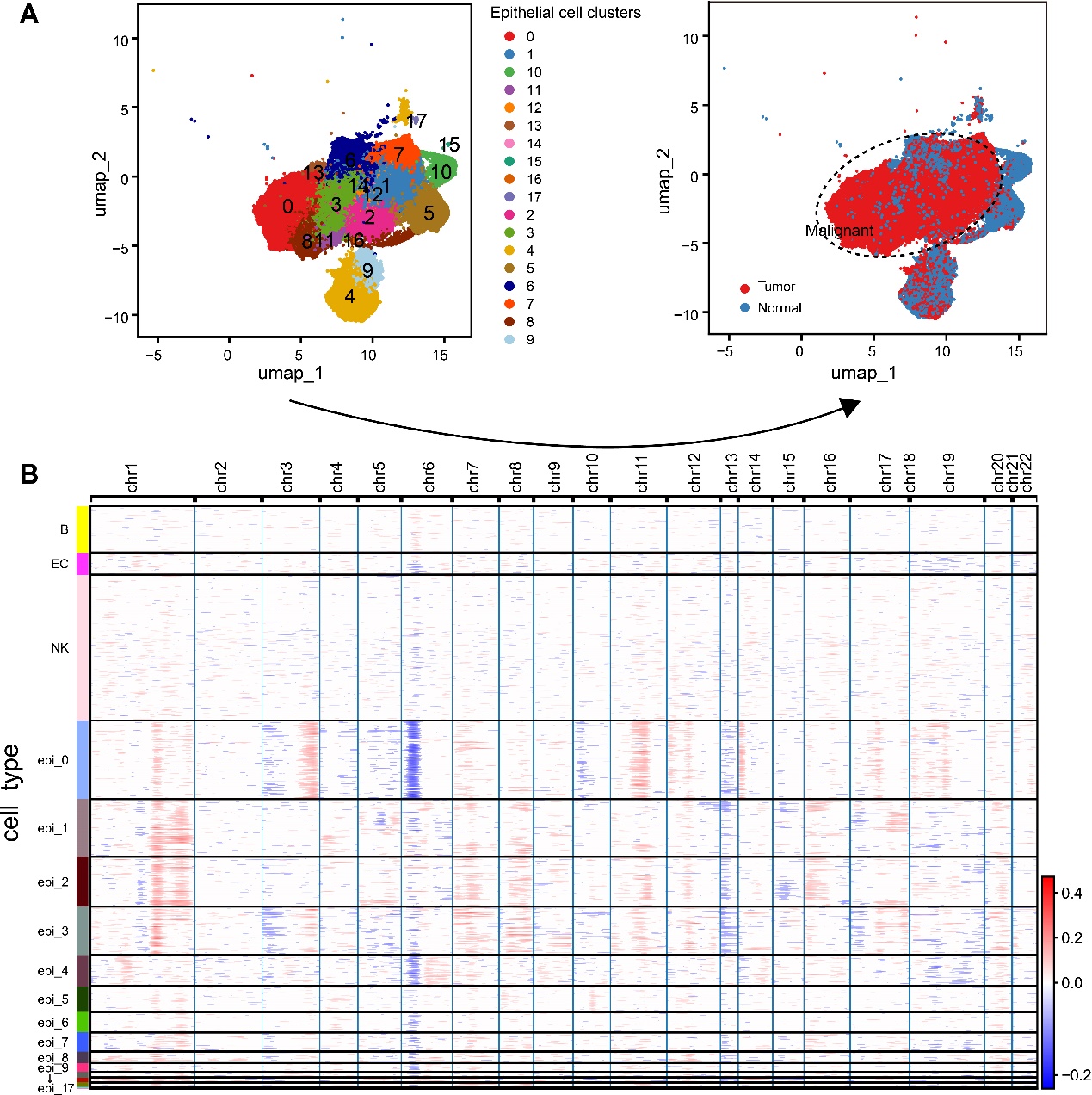


**Supplementary Figure 2**

**
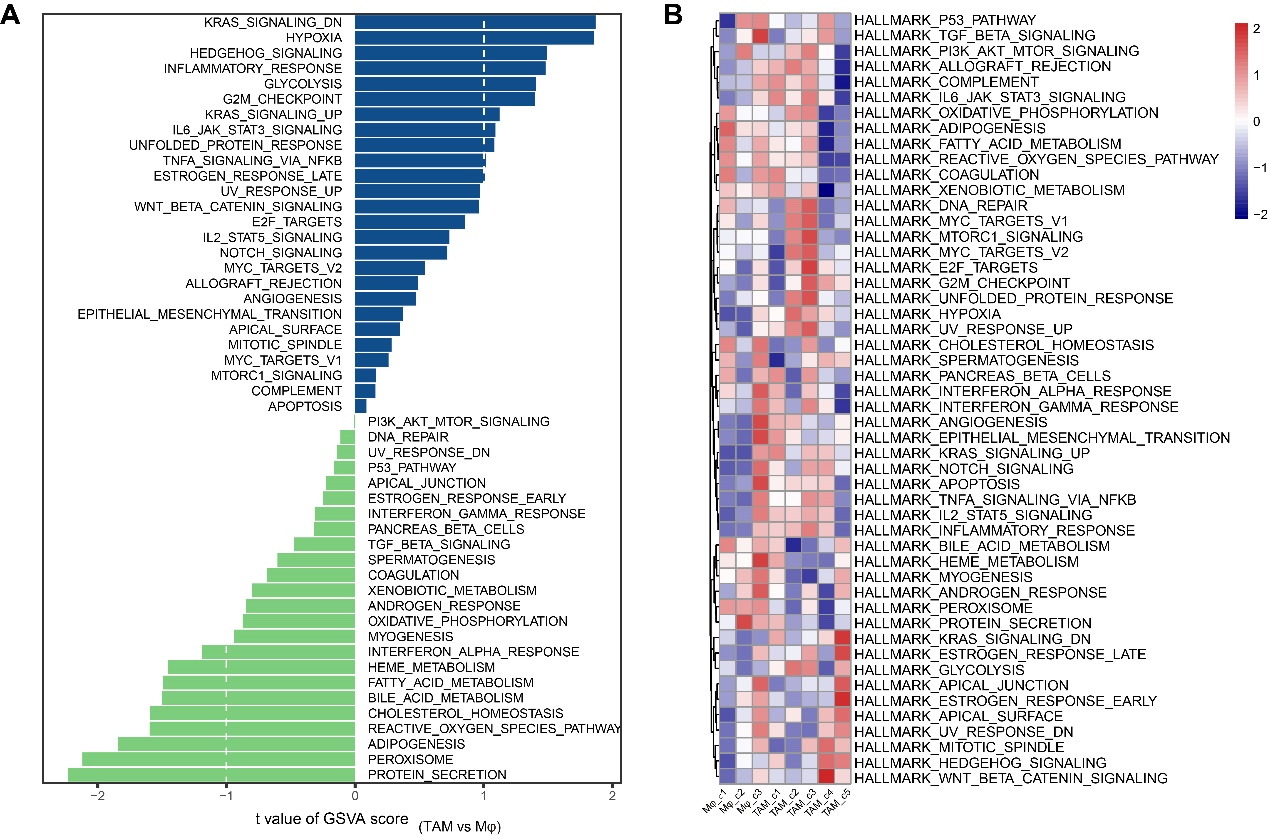
**

**Supplementary Figure 3**

**
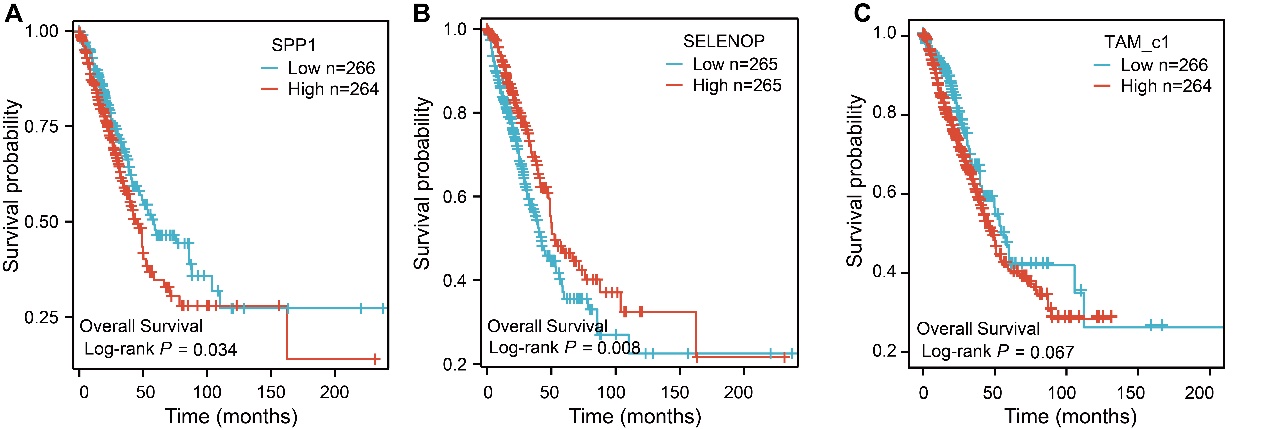
**

**Supplementary Figure 4**

**
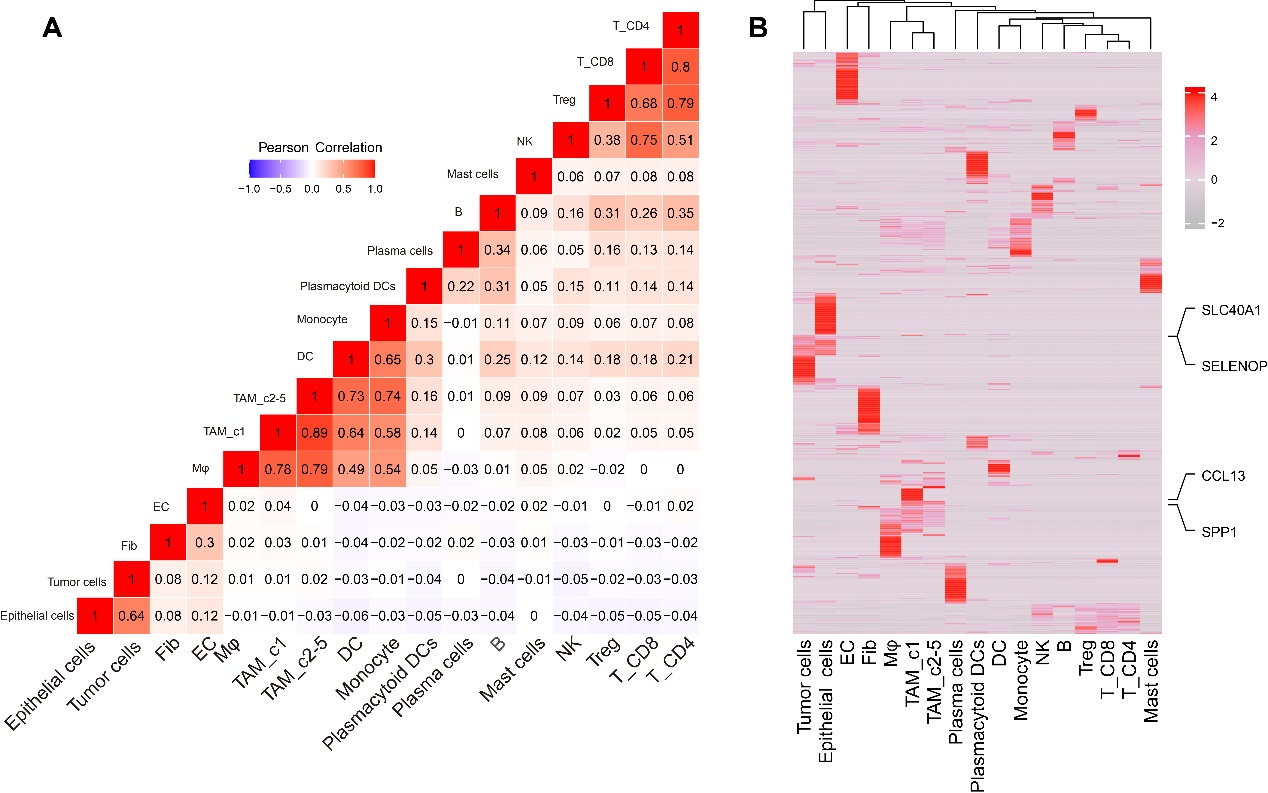
**

Supplement: Supplementary file 2 [file DataSheet1.docx]
